# Knockdown of SMAD3 inhibits the growth and enhances the radiosensitivity of lung adenocarcinoma via p21 *in vitro* and *in vivo*

**DOI:** 10.7150/ijbs.40173

**Published:** 2020-01-30

**Authors:** Hao Niu, Yiwei Huang, Li Yan, Li Zhang, Mengnan Zhao, Tao Lu, Xiaodong Yang, Zhengcong Chen, Cheng Zhan, Yu Shi, Qun Wang

**Affiliations:** 1 Department of Radiation Oncology, Zhongshan Hospital, Fudan University, Shanghai, China.; 2 Department of Thoracic Surgery, Zhongshan Hospital, Fudan University, Shanghai, China.; 3 Department of Radiation Oncology, Eye & ENT Hospital, Fudan University, Shanghai, China.

**Keywords:** SMAD3, radio-sensitivity, lung adenocarcinoma, cell cycle, p21, prognosis

## Abstract

Radiotherapy is an effective approach for the treatment of lung adenocarcinoma. However, evidence suggests that lung adenocarcinoma can easily develop tolerance to radiotherapy. The purpose of this study was to investigate the effect and mechanism of SMAD3 on the radiosensitivity of lung adenocarcinoma *in vitro* and *in vivo*. We found that knockdown of SMAD3 using two short hairpin RNAs in lentivirus vectors significantly inhibited cell growth and increased radiosensitivity of the lung adenocarcinoma cell lines A549, H1299, and H1975. Using RNA sequencing and bioinformatics analyses, we found that the significantly differentially expressed genes in SMAD3 knockdown cells were mainly enriched in the cell cycle process. We then showed that knockdown of SMAD3 significantly reduced expression of cyclin-dependent kinase inhibitor 1 (p21) and increased the proportion of G2/M phase cells and the radiosensitivity of lung adenocarcinoma. Chromatin immunoprecipitation results in the Gene Expression Omnibus (GEO) database and our luciferase assay verified that SMAD3 directly bound the p21 promoter. A series of rescue experiments showed that overexpression of p21 partly reversed the effect of SMAD3 on proliferation and radioresistance *in vitro* and *in vivo*. Moreover, we found that the expression levels of SMAD3 and p21 were highly correlated, and both correlated with the patients' survival in online databases and clinical specimens. Expression of SMAD3 and p21 was also significantly different between radioresistant and radiosensitive patients in our hospital. Our results indicate that SMAD3 is a potential prognosis and radiosensitivity indicator as well as a target for radiotherapy and other treatments of patients with lung adenocarcinoma.

## Introduction

Lung cancer is still the leading cause of cancer incidence and mortality, with 2.1 million new cases and 1.8 million deaths in 2018, representing 18.4% of cancer deaths 1. The 5-year survival rate of lung cancer is low, ranging from 3.7-33.7% in different countries in 2010-2014 2. Adenocarcinoma is a major subtype of lung cancer, and its proportion continues to increase 3. According to the National Comprehensive Cancer Network guidelines, radiotherapy plays a major role in lung adenocarcinoma patients for whom surgery is no longer an option. It is also frequently used in adjuvant and neoadjuvant therapy of patients with advanced lung adenocarcinoma. Stereotactic body radiation therapy has recently been recommended as the most favorable therapy choice besides surgery for early stage adenocarcinoma, and is also considered for the treatment of local or distant metastases 4.

However, lung adenocarcinoma is not highly sensitive to radiation, and the progressive tolerance of tumor cells during irradiation often causes local recurrence and poor prognosis 5. Because the sensitivity of tumor cells is a key factor in the efficacy of radiotherapy, it is necessary to identify critical molecules related to the radiosensitivity of lung adenocarcinoma, as well as the factors linked to rapid cell growth. Here, we report that the transcription factor SMAD3 inhibited cell growth and promote radiosensitivity of lung adenocarcinoma both *in vitro* and *in vivo* via p21/cell cycle. We believe that our findings will help improve the efficacy of radiotherapy and patients' survival.

## Methods and Materials

### Cell culture and lentivirus infection

Lung adenocarcinoma cell lines (A549, H1299, and H1975) were purchased from the Chinese Academy of Sciences Cell Bank. Cells were cultured in RPMI1640 (Hyclone, Logan, UT, USA) supplemented with 10% fetal bovine serum (Hyclone), 100 U/mL penicillin, and 100 U/mL streptomycin in a humidified 5% CO_2_ atmosphere at 37°C. Two different short hairpin RNAs (shRNAs) targeting SMAD3 were designed and cloned into a lentivirus vector with puromycin resistance, which was transfected into cells and screened using puromycin. Sequences of the shRNAs and the control are provided in Table Supplementary 1 (Table S1).

### RNA sequencing

Total RNA from cells transfected with SMAD3-NC, SMAD3-SH1, and SMAD3-SH2 were processed by oligo dT enrichment (removal of rRNA) and the library was constructed using the KAPA Stranded RNA-Seq Library Prep Kit (Shanghai Yihui Biological Technology Co., Ltd., Shanghai, China). The mixed sample libraries were sequenced using an Illumina HiSeq 4000 sequencer. Image processing and base recognition were performed using Solexa pipeline version 1.8 (Off-Line Base Caller, version 1.8) software. Differentially expressed genes were annotated and analyzed by R package DESeq. And the significant genes were chosen with padj<0.05. The Kyoto Encyclopedia of Genes and Genome (KEGG) pathways annotation was conducted by R packages, including Rgraphviz, pathview, clusterProfiler,and org.Hs.eg.db. The enrichment was call differentially enriched if they exhibited a Corrected *P*-value<0.05.

### Quantitative real-time polymerase chain reaction (qRT-PCR)

PrimeScript RT Reagent Kit (TaKaRa, Tokyo, Japan) was used to synthesize cDNA template and SYBR Premix Ex Taq (Takara) was used to perform qRT-PCR analysis. All reactions were analyzed in an Applied Biosystems system 7500 (Thermo Fisher Scientific, Waltham, MA, USA). Relative quantification of mRNA was calculated using the 2^-ΔΔCT^ method using β-ACTIN as an endogenous calibrator. All primers were synthesized by Sangon Biotech (Shanghai, China) and their sequences are provided in Table Supplementary 2 (Table S2).

### Western blotting

Proteins were extracted from cells using RIPA buffer (Beyotime, Shanghai, China) with protease and phosphatase inhibitor cocktail (Beyotime) and quantified using an Enhanced BCA Protein Assay Kit (Beyotime). Proteins were then separated by SDS-PAGE and transferred onto polyvinylidene fluoride membranes (Merck-Millipore, Burlington, MA, USA) (Constant current 0.32A, 90 mins). The membranes were blocked with 5% nonfat milk for 1h and then incubation with specific primary antibodies for 12h at 4°C. Finally, the protein bands were visualized by Moon Chemiluminescence Reagent kit (Beyotime). The following antibodies were used: anti-SMAD3 (1:1000, #9523, Cell Signaling Technology, Danvers, MA, USA), anti- phospho-SMAD3 (1:1,000, Ser423/425), p-SMAD3 antibody (1:1,000, #9520, Cell Signaling Technology), anti-p21 (1:1,000, #2947, Cell Signaling Technology), anti-β-ACTIN (1:3,000, AA128, Beyotime), horseradish peroxidase (HRP)-labeled goat anti-rabbit IgG (H+L) (1:5,000, A0208, Beyotime), and HRP-labeled goat anti-mouse IgG (H+L) (1:5,000, A0208, Beyotime).

### Cell cycle assay

Briefly, cells were harvested at 80-90% confluence and stained with 0.1 mg/mL propidium iodide. The cell cycle was detected by LSR Fortessa flow cytometry (BD Biosciences, San Jose, CA, USA).

### Cell proliferation assay

A total of 2,000 cells at logarithmic growth phase were seeded into black 96-well plates (Life Science, Oneonta, NY, USA) at 100 uL of cell suspension per well. Following incubation for 0, 24, 48, 72, 96, and 120 h at 37°C, cell proliferation was measured using a Enhanced Cell Counting Kit-8 Viability Assay Kit (Beyotime).

### Clone formation assay

Cells were seeded in triplicate into 6 cm plates at a density of 800 cells/plate at logarithmic growth phase. After incubation for 24 h, cells were irradiated with X-ray (0, 2, 4, 6, 8, and 10 Gy). Irradiation process: 1.5- cm compensation film was placed under the cell culture dish. Cells were irradiated with a gradient dose (0, 2, 4, 6, 8, and 10 Gy) using an ONCOR™ linear accelerator (photon beam energy of 6 MV, dose rate of 300 cGy/min) with a gantry angle rotated to 180 ° and a source surface distance of 100 cm. Then, cells were maintained for 10-14 days, fixed with 4% methanol, and stained with 1% purple crystal. Viable colonies with a diameter of ≥ 0.2 mm were counted.

### Dual-luciferase reporter assay

The potential binding sites of SMAD3 in the p21 promoter (from -1000 bp to +200 bp) were predicted using the JASPAR database 6. The regions of the p21 promoter and corresponding mutations were subcloned into the pGL3-basic luciferase reporter plasmid vector (Promega, Madison, WI, USA). Then, the constructed vectors or Renilla pRLTK plasmid (Promega) and SMAD3 overexpression (SMAD3-OE) or control plasmid were transiently co-transfected into 293T cells at 70-80% confluence using Lipo8000 (Beyotime) in 24-well plates. After transfection for 48 h, Dual-Lumi™ Luciferase Reporter Gene Assay Kit (Beyotime) was used to detect luciferase activity according to the manufacturer's instructions. Each experiment was done in six repetitions. The results are shown as firefly luciferase activity normalized to Renilla luciferase activity.

### Subcutaneous tumor formation

Male BALB/c nude mice (6-weeks-old) were purchased from Shanghai Slac Laboratory Animal Co., Ltd. and maintained in laminar flow cabinets under specific pathogen free conditions. Nude mice were subcutaneously inoculated with 1 × 10^6^ A549 cells. After 14 days, part of the nude mice were exposed to three doses of 6 Gy X-rays with an interval of 3 days^7^, ^8^. The mice were sacrificed 4 weeks after inoculation and the tumors were weighed for further analysis.

The mice were divided into four major groups as follows: Group 1: knockdown of SMAD3 without irradiation (SMAD3 negative control [SMAD3-NC], SMAD3-SH1, and SMAD3-SH2); Group 2: knockdown of SMAD3 with irradiation (SMAD3-NC + RT, SMAD3-SH1 + RT, and SMAD3-SH2 + RT); Group 3: knockdown of SMAD3 and co-transfection with p21 overexpression vector (SMAD3-NC + p21, SMAD3-SH1 + p21, and SMAD3-SH2 + p21) without irradiation; and Group 4: knockdown of SMAD3 and co-transfection with p21 overexpression vector (SMAD3-NC + p21 + RT, SMAD3-SH1 + p21 + RT, and SMAD3-SH2 + p21 + RT).

### Patients and specimens

Tissue specimens were obtained from 119 patients who received surgery between February 2014 and December 2018 (30-75 years old) and were histologically diagnosed with lung adenocarcinoma. The patients' survival was extracted from follow-up records. We follow up the patients every six months. The missing patients were recorded as missing data. If the patient died, the follow-up was terminated.

### Immunohistochemistry

Expression of SMAD3 and p21 was detected using GTvision^TM^ HRP-polymer anti-mouse/rabbit IHC Kit (GeneTech, Shanghai, China). Rabbit anti-human anti-SMAD3 (1:500, #9523, Cell Signaling Technology) and rabbit anti-p21 (1:100, #2947, Cell Signaling Technology) antibodies were used. The results were independently judged by two researchers.

Two physicians independently judged whether staining according to the immunoreactivity score (IRS), which was calculated as the staining intensity score multiplied by the proportion of positive cells. The criterion for judging staining intensity was as follows: colorless (negative), 0; light yellow (weak), 1; dark yellow (moderate), 2; and yellowish brown (strong), 3. The standard was judged by comparing the colored cells with the colored background. The criterion for the proportion of positive cells was as follows: 0%, 0; 1-30%, 1; 31-60%, 2, and 61-100%, 3. For each tissue, IRS < 4 was considered as low expression and IRS ≥ 4 was considered as high expression.

### Statistical analysis

Graphpad Software (5.0), R software (3.63), and SPSS software (20.0) were used to assess data. Student's *t*-test was used to compare the differences between two groups. Single target multihit model was used to further measure the radiosensitivity of cells. Kaplan-Meier survival curves and log-rank test were employed to depict overall survival (OS). Correlations between two variations were analyzed by Pearson's correlation. Two GEO datasets (GSE102225 and GSE 130364) were used to investigate the regulatory effect of SMAD3 on p21 in other cell types and SMAD3 chromatin immunoprecipitation (ChIP)-Seq results. GEPIA (based on TCGA and GTEx data) (http://gepia.cancer-pku.cn/index.htmL) 9 and Kmplot (http://kmplot.com/analysis/index.php?p=service&cancer=lung) 10 were used to analyze the relationship between SMAD3 or p21 and the prognosis of patients with lung adenocarcinoma. The correlation between SMAD3 and p21 was also analyzed with GEPIA and KMplot. The statistical significances of groups are represented as ^*^P < 0.05, ^**^P < 0.01, and ^***^P < 0.001.

## Results

### Knockdown of SMAD3 inhibits the growth and promotes the radiosensitivity of lung adenocarcinoma cells

**Figure 1:**
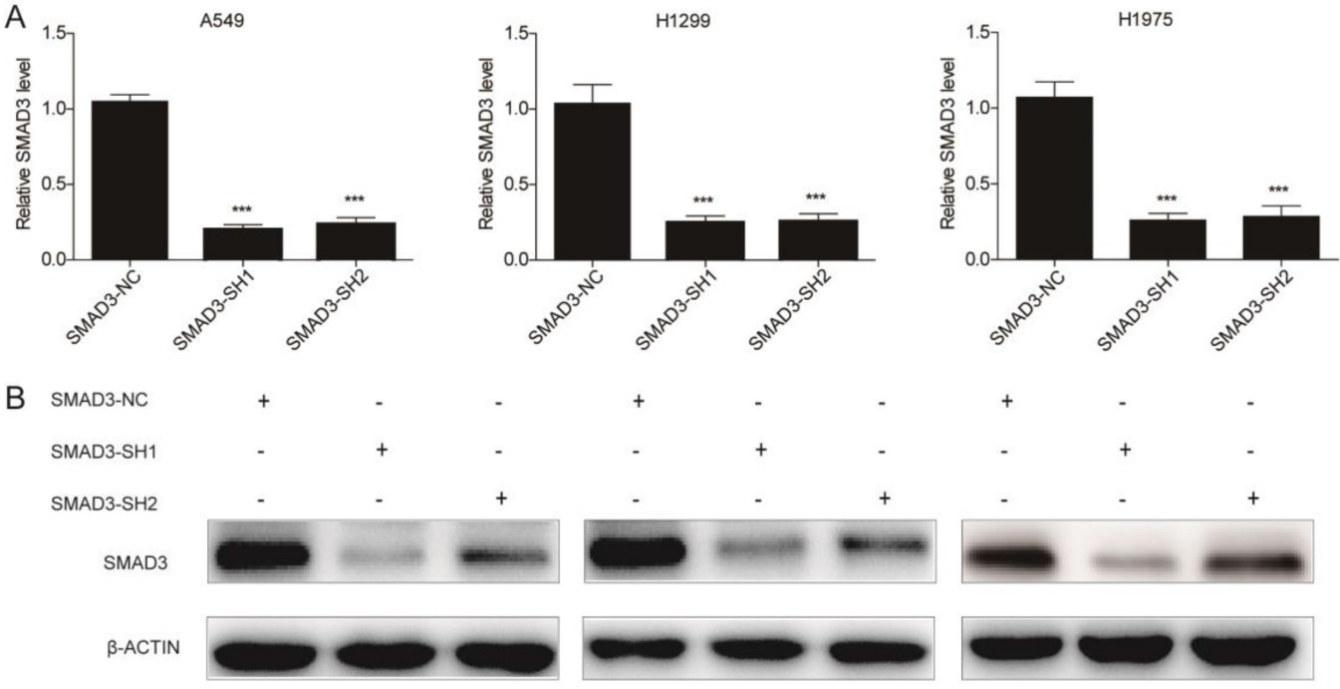
** Expression of SMAD3 was significantly decreased in A549, H1299, and H1975 cells transfected with short hairpin RNA (shRNA) targeting SMAD3. (A)** Quantitative RT-PCR and **(B)** western blotting analyses of the expression of SMAD3 in A549, H1299, and H1975 cells infected with SMAD3-NC, SMAD3-SH1, and SMAD3-SH2. NC, negative control. SH1, short hairpin 1. SH2, short hairpin 2. Data are presented as mean ± SEM. ^*^P < 0.05, ^**^P < 0.01, and ^***^P < 0.001. SEM, standard error of mean.

To explore the role of SMAD3 in non-small cell lung cancer, we designed two different shRNAs targeting SMAD3, and transfected them into three lung adenocarcinoma cell lines to knockdown SMAD3 expression. Expression of SMAD3 was significantly decreased in the SMAD3-SH1 and SMAD3-SH2 groups compared with SMAD3-NC by both qRT-PCR and western blotting (**Fig. 1A, 1B**).

**Figure 2:**
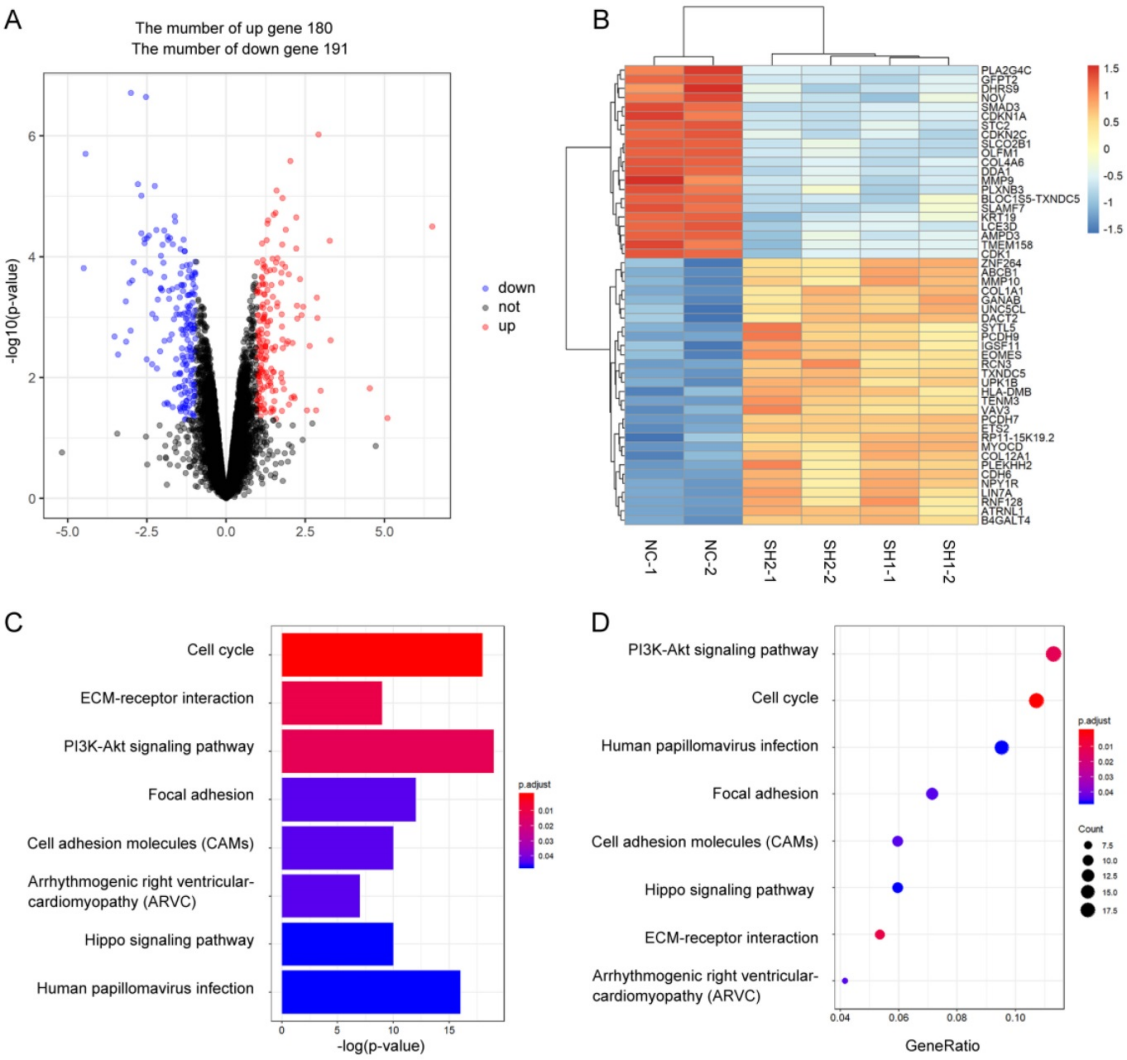
** Identification the changes in cellular pathways and related gene expression levels after knockdown of SMAD3 gene in A549 lung cancer cell lines. (A)** Volcano plot showed the differentially expressed mRNAs in knockdown of SMAD3 in A549 cells compared to the negative control in A549 cells. The red dots or the blue dots represent the mRNAs increasing or decreasing fold-changes >2.0 and *P*‐values <0.05, respectively. **(B)** The heatmap shows the top 50 genes with the most significant changes in gene expression levels in the A549 cell line after SMAD3 knockdown. **(C)** As determined by the Kyoto Encyclopedia of Gene and Genomes (KEGG) Pathways analysis, the size and color represent the range of *p* values. **(D)** Dot plot of enriched pathways is indicated as the gene-ratio of the differentially expressed gene number to the total gene number in a certain KEGG annotation.

**Figure 3:**
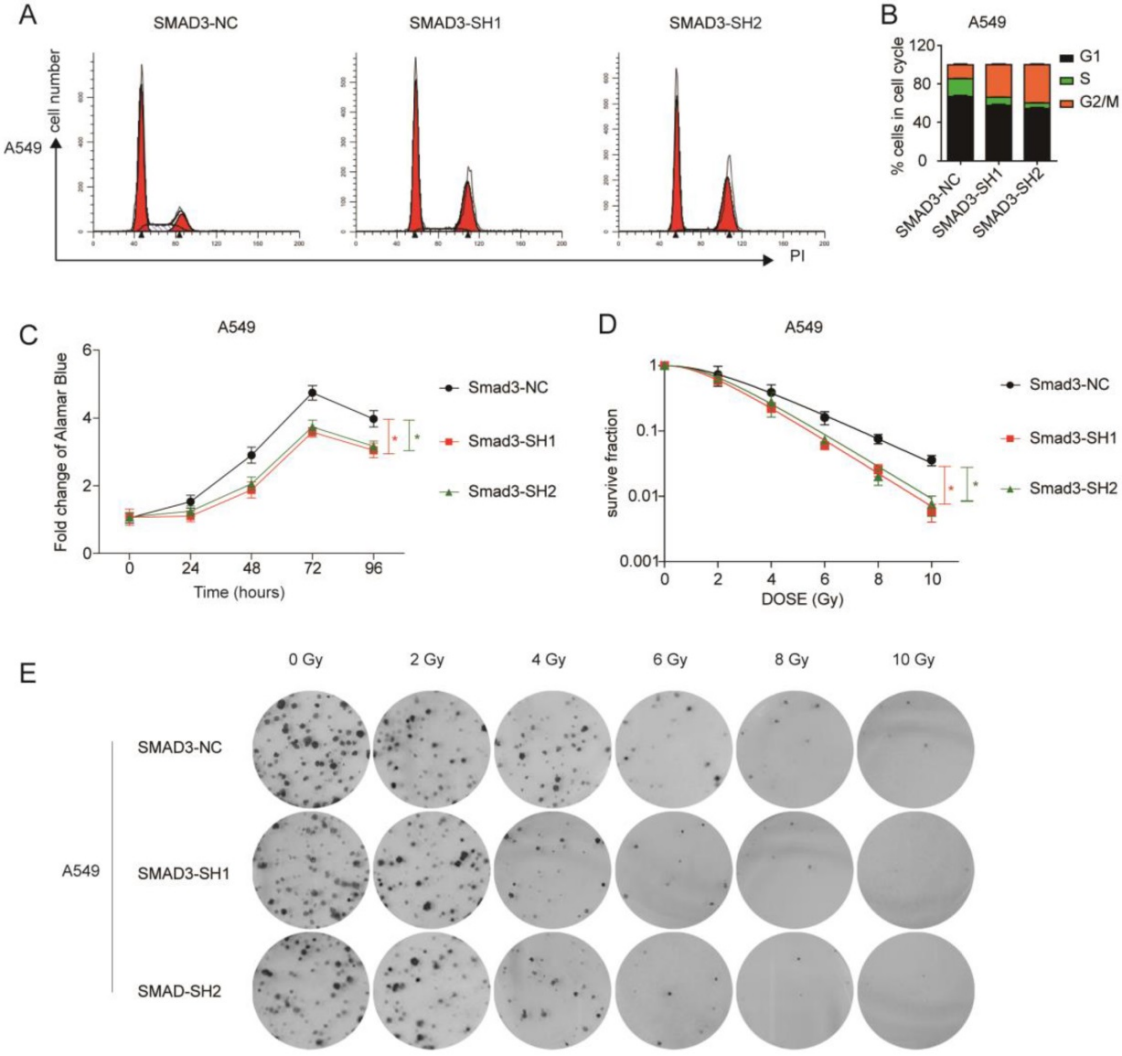
** Knockdown of SMAD3 enhanced radiosensitivity and inhibited the proliferation of lung adenocarcinoma cells to irradiation. (A, B)** Knockdown of SMAD3 significantly increased the proportion of G2/M phase in A549 cells detected by flow cytometry. **(C)** Knockdown of SMAD3 significantly inhibited the proliferation of A549 cells. **(D, E)** The number colonies of lung adenocarcinoma cells was significantly decreased following knockdown of SMAD3 in A549 cells, as analyzed by clone formation assay and single target multi-shot model curve fitting. Data are presented as mean ± SEM. *P < 0.05, **P < 0.01, and ***P < 0.001. SEM, standard error of mean.

To explore the role of SMAD3 in lung adenocarcinoma, we detected changes in gene expression following SMAD3 knockdown in A549 cells using RNA-Seq. The volcano plot demonstrated that 180 genes were significantly up and 191 genes were significantly down (fold‐changes >2.0 and P‐values <0.05) in response to knockdown of SMAD3 in A549 cells (**Fig.2A**). The heatmap shows the top 50 genes with the most significant changes in gene expression levels in the A549 cell line after SMAD3 knockdown (**Fig. 2B**). The top 371 significantly different genes were analyzed using KEGG pathway, which indicated that 'Cell cycle' pathways ranked the top mapped pathways and the second pathways as analyzed with the range of p values and gene-ratio, respectively (**Fig. 2C, 2D**). Knockdown of SMAD3 also significantly increased the proportion of G2/M phase cells (**Fig. 3A, 3B, Fig. S1A, S1B**). Cell proliferation assays demonstrated that knockdown of SMAD3 dramatically inhibited the proliferation of A549, H1299, and H1975 cells (**Fig. 3C, Fig. S1C**). Clone formation assay was performed to investigate changes in the radiosensitivity of cells following knockdown of SMAD3. The results showed that the numbers of lung adenocarcinoma cell colonies decreased following doses of 2, 4, 6, 8, and 10 Gy X-ray compared with the 0 Gy X-ray group. Moreover, the number of lung adenocarcinoma cell colonies was significantly decreased compared with SMAD3-NC by single target multi-shot model curve fitting (**Fig. 3D, 3E, Fig. S1D, S1E**). These results indicated that knockdown of SMAD3 enhanced radiosensitivity of lung adenocarcinoma cells.

### Interaction between SMAD3 and p21

Of the differentially expressed genes enriched in cell cycle regulation, cyclin-dependent kinase inhibitor 1 A (p21), a fundamental regulator of the cell cycle, was significantly decreased in SMAD3 knockdown cells. We checked the GEO database (GSE102225) and found that p21 was significantly decreased in SMAD3 knockdown TPC-1 cells, a thyroid cancer cell line, further confirming the regulatory effect of SMAD3 on p21 11. Although p21 has been frequently reported as a downstream target of SMAD3, the regulatory mechanism has not yet been elucidated.

**Figure 4:**
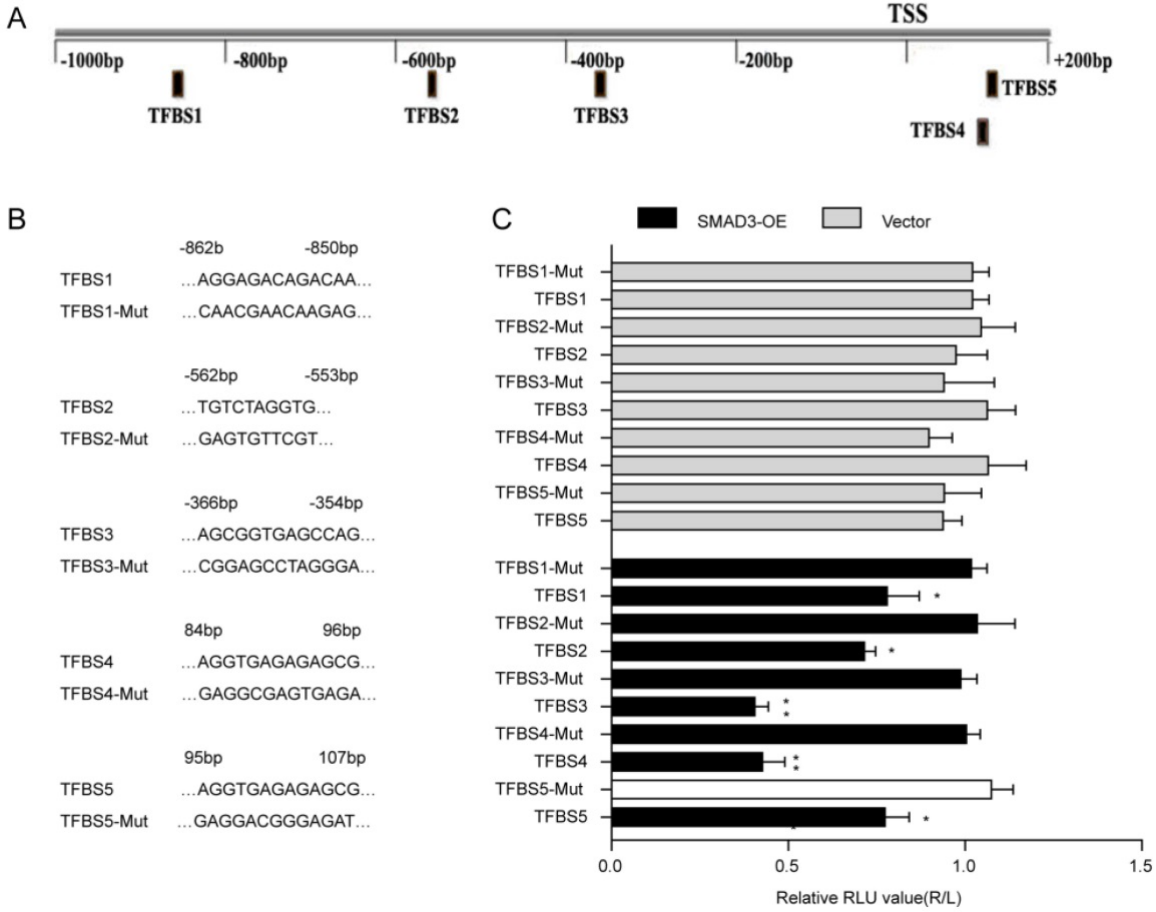
** Interaction between SMAD3 and p21. (A)** Genes that interact with SMAD3 and binding regions were predicted using the JASPAR database. **(B)** Five transcription factor binding sites (TFBSs) of p21 with good score and corresponding mutation were subcloned into the luciferase reporter plasmid vector. **(C)** Co-transfection of the TFBS3-WT or TFBS4-WT plasmid with SMAD3-WT vector significantly decreased luciferase activity using a dual-luciferase reporter assay. WT: wild type. Data are presented as mean ± SEM. ^*^P < 0.05, ^**^P < 0.01, and ^***^P < 0.001. SEM, standard error of mean.

The JASPAR database was used to predict the binding sites of SMAD3 in the p21 promoter(**Fig. 4A**). Five transcription factor binding sites (TFBSs) of p21 with good score and corresponding mutation were subcloned into the luciferase reporter plasmid vector(**Fig. 4B**). Results of the dual-luciferase reporter assay indicated that luciferase activity was significantly decreased with co-transfection of TFBS3 or TFBS4 of the p21 promoter with the SMAD3-WT vector group compared with co-transfection of truncated regions of the p21 promoter mutation with the SMAD3-NC vector (**Fig. 4C**). ChIP-Seq results obtained from GSE130364 also revealed that SMAD3 bound the promoter region of p21 in breast cancer cells. These results indicated that SMAD3 directly bound the p21 promoter and regulated the transcription of p21.

### Overexpression of p21 reversed the effects of SMAD3 on cell proliferation and radiosensitivity in lung adenocarcinoma cells

**Figure 5:**
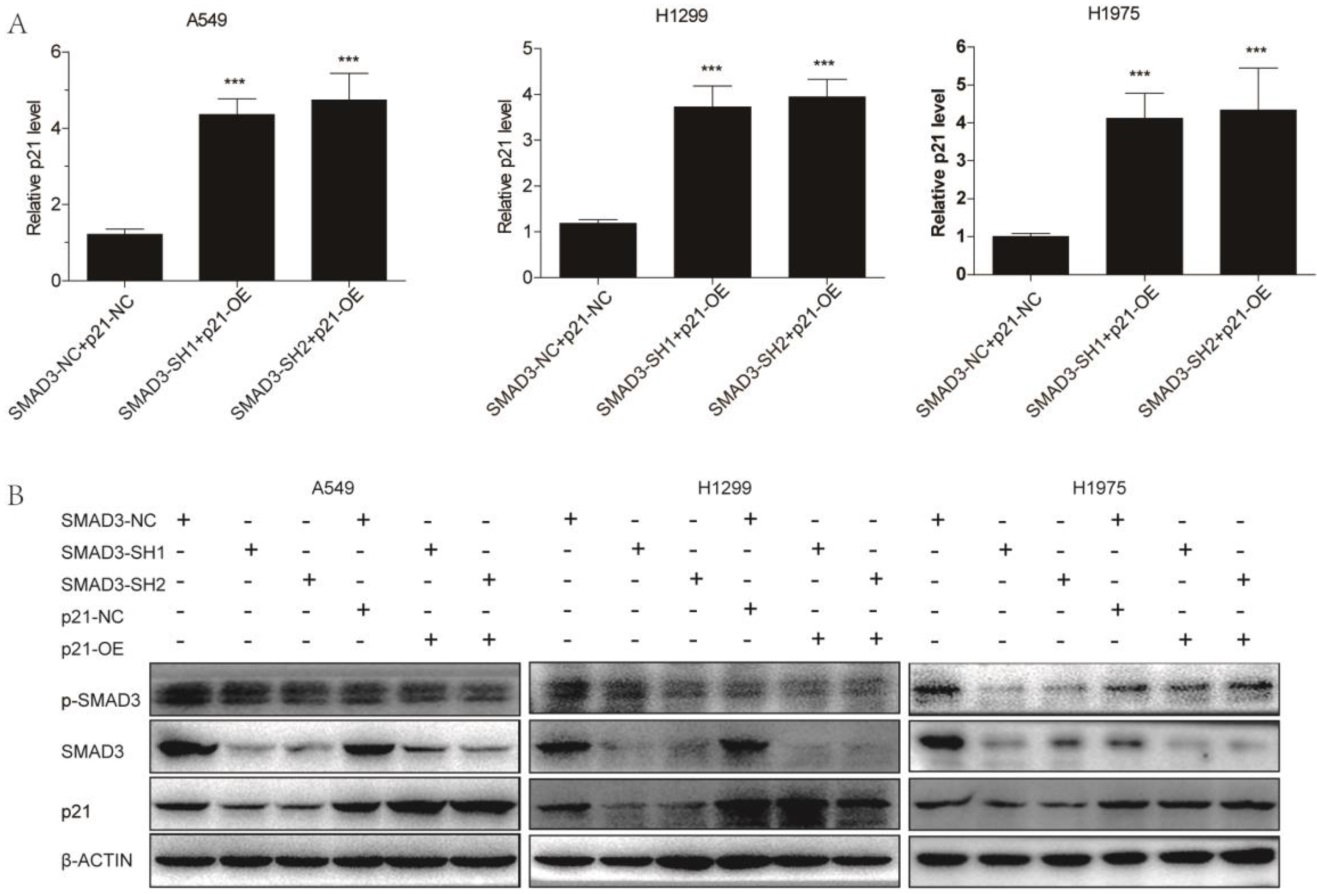
** Overexpression of p21. (A)** Quantitative RT-PCR revealed the efficience of overexpression of p21 in A549, H1299, and H1975 cells infected with shRNA targeting SMAD3. **(B)** Western blotting showed that overexpression of p21 partly reversed the decrease in p21 protein levels caused by knockdown of SMAD3 in A549, H1299, and H1975 cells. Overexpression of p21 did not affect SMAD3 and p-SMAD3 protein levels in A549, H1299, and H1975 infected with shRNA targeting SMAD3. shRNA, short hairpin RNAs. Data are presented as mean ± SEM. *P < 0.05, **P < 0.01, and ***P < 0.001. SEM, standard error of mean.

**Figure 6:**
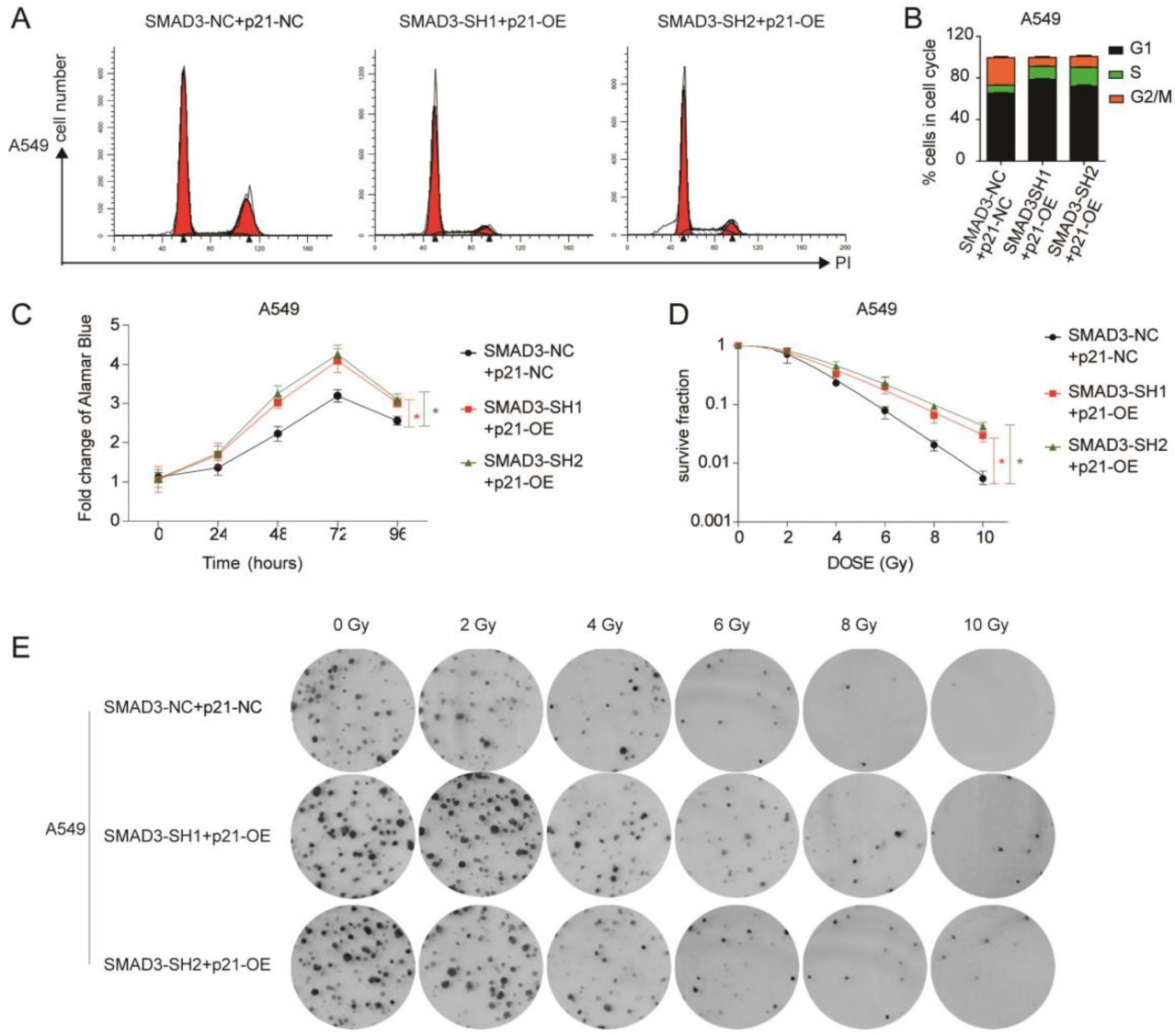
** Overexpression of p21 partly reversed the effect of knockdown of SMAD3 in lung adenocarcinoma cells. (A, B)** Cell cycle analysis demonstrated that overexpression of p21 significantly decreased the percentage of G2/M phase cells in A549 cells infected with shRNA targeting SMAD3 by flow cytometry. **(C)** Overexpression of p21 reversed the inhibition of proliferation caused by knockdown of SMAD3 in A549 cells. **(D, E)** Analysis of clone formation assay by single target multi-shot model curve fitting demonstrated that the number of cell colonies was significantly increased by overexpression of p21 in A549 cells infected with shRNA targeting SMAD3 at the same dose of irradiation. shRNA, short hairpin RNAs. Data are presented as mean ± SEM. *P < 0.05, **P < 0.01, and ***P < 0.001. SEM, standard error of mean.

We overexpressed p21 using lentivirus in SMAD3 knockdown cells, and assessed whether it could rescue the effects of SMAD3 knockdown. qRT-PCR and western blotting demonstrated that the expression of p21 was significantly downregulated in SMAD3 knockdown cells, but its expression was reversed in cells with p21 overexpression (p21-OE) (**Fig. 5A, 5B**). Cell cycle analysis demonstrated that overexpression of p21 also significantly reduced the percentage of SMAD3 knockdown cells in G2/M phase (**Fig. 6A, 6B, Fig. S2A, S2B**). This indicated that overexpression of p21 reversed the increase in G2/M phase cells induced by knockdown of SMAD3. The results of the cell proliferation assay demonstrated that overexpression of p21 reversed the inhibition of proliferation caused by SMAD3 knockdown in all three lung adenocarcinoma cell lines (**Fig. 6C, Fig. S2C**). Furthermore, clone formation assay increased with overexpression of p21 compared with control cells with the same dose of irradiation (**Fig. 6D, 6E Fig. S2D, S2E**). These results provided strong evidence that overexpression of p21 reversed the enhancing radiosensitivity induced by knockdown of SMAD3 in lung adenocarcinoma cells.

### Knockdown of SMAD3 inhibits the growth and promotes the radiosensitivity of A549 cells via p21 in a mouse model

**Figure 7:**
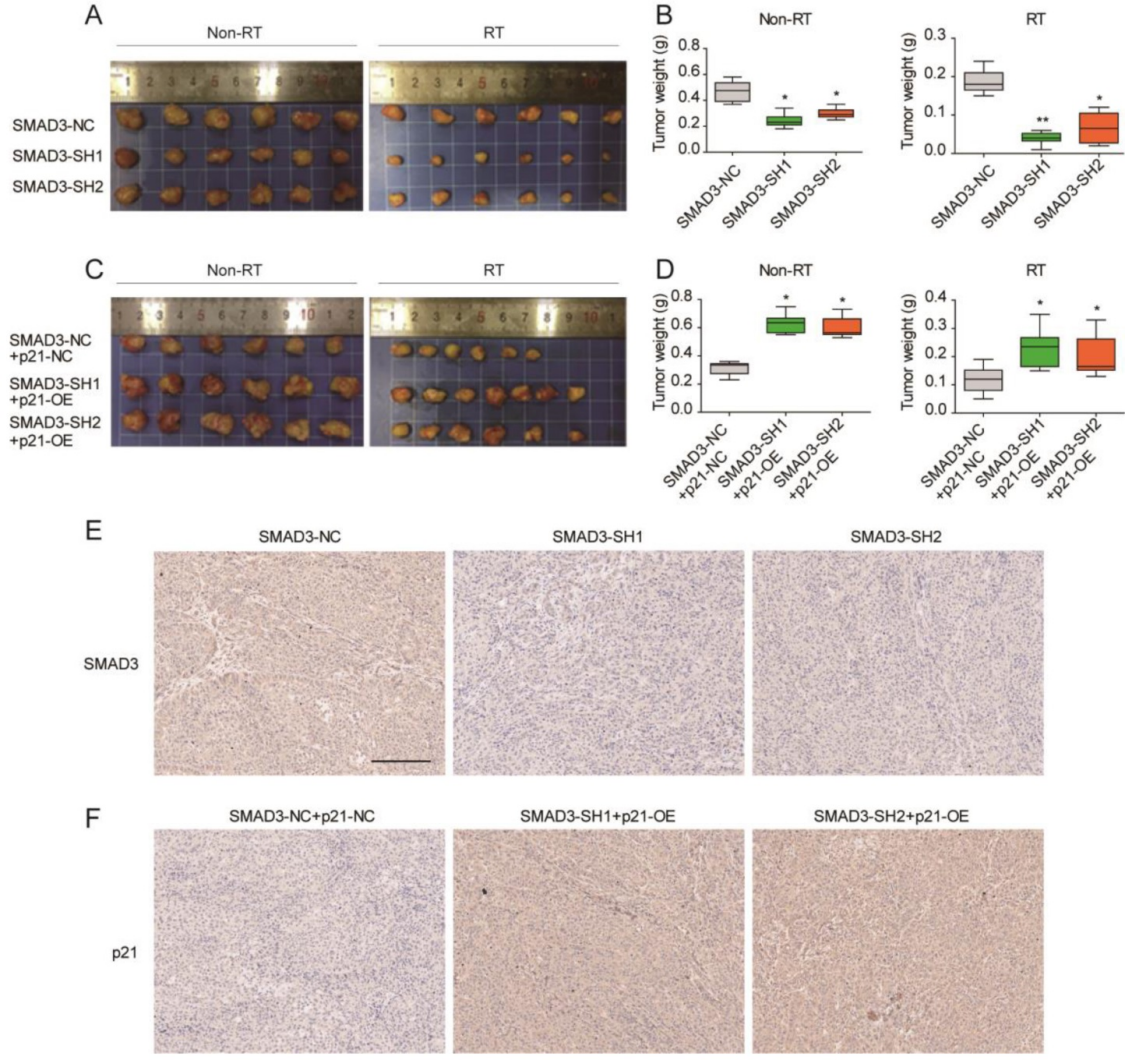
** Knockdown of SMAD3 inhibits the growth and promotes the radiosensitivity of A549 cells via p21 in a mouse model. (A, B)** Knockdown of SMAD3 inhibited tumor growth and enhanced tumor radiosensitivity to radiation *in vivo*. **(C, D)** Overexpression of p21 promoted tumor growth and reduced tumor radiosensitivity to radiation *in vivo*. **(E)** Immunohistochemistry confirmed that the expression of SMAD3 was significantly decreased in knockdown of SMAD3 tumor tissues and p21 was significantly increased in overexpression p21 tumor tissues. Scale bar, 200 μm. Data are presented as mean ± SEM. *P < 0.05, **P < 0.01, and ***P < 0.001. SEM, standard error of mean.

To verify the effect of SMAD3 on the radiosensitivity and its regulatory mechanism *in vivo*, a subcutaneous tumor formation assay was conducted in nude mice. Compared with non- irradiation groups, the irradiation groups results confirmed that irradiation inhibited tumor growth (**Fig. 7A- 7D**). Knockdown of SMAD3 inhibited tumor growth and promoted radiosensitivity of A549 cells in the mouse model (**Fig. 7A, 7B**). Overexpression of p21 promoted tumor growth and enhanced tumor radioresistance to irradiation *in vivo* (**Fig. 6B, 6C**). In short, p21 overexpression reversed the effect of SMAD3 on cell growth and the radiosensitivity of lung adenocarcinoma cells *in vivo*, which was consistent with the *in vitro* results. Immunohistochemistry confirmed that the expression of SMAD3 was significantly decreased in knockdown of SMAD3 tumor tissues and p21 was significantly increased in overexpression p21 tumor tissues. Furthermore, we also found that expression levels of SMAD3 and p21 were not statistically different before or after irradiation.

### Correlation among SMAD3 expression, survival, and radiosensitivity in patients with lung adenocarcinoma

**Figure 8:**
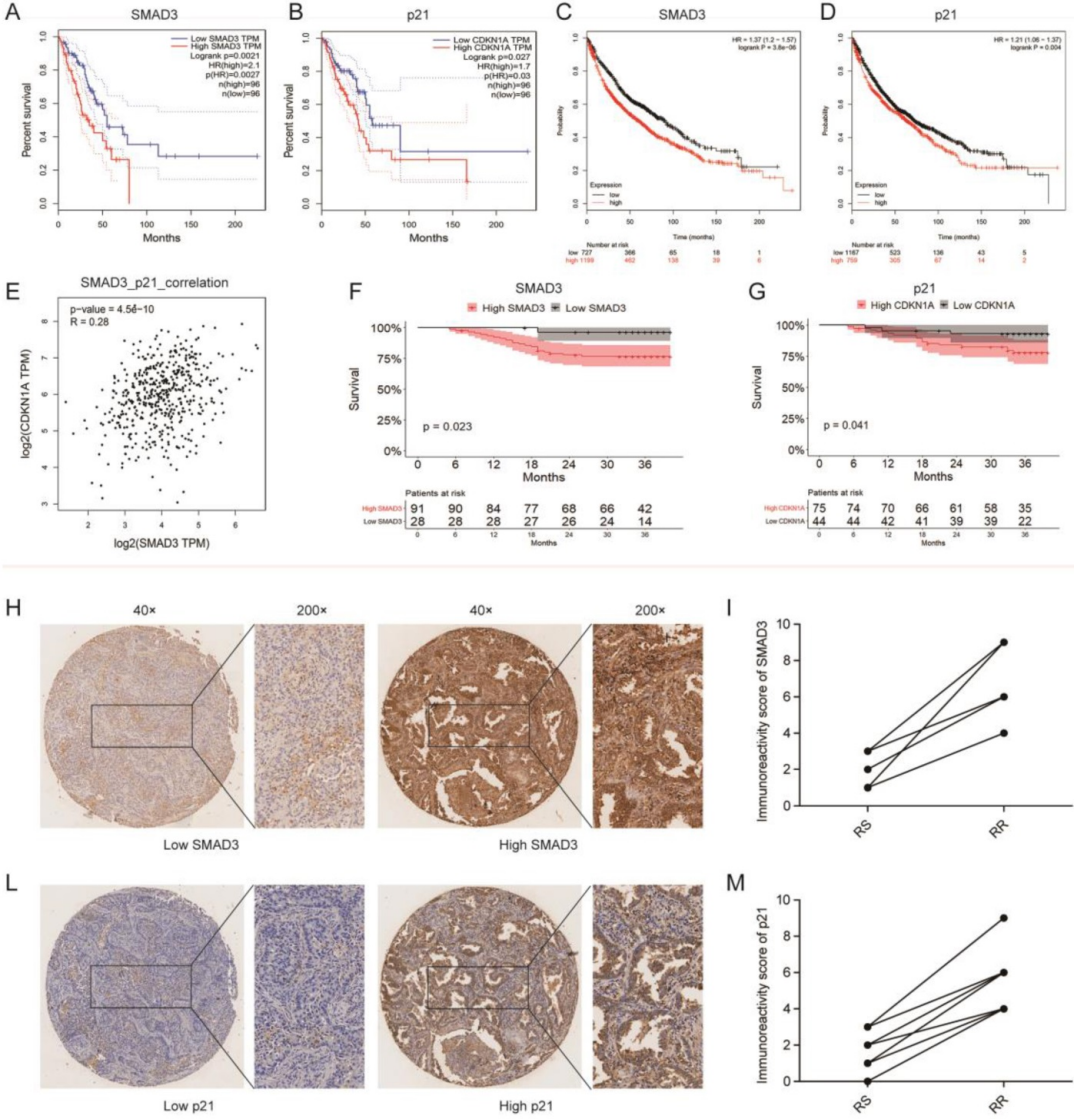
** Correlation among SMAD3 expression, survival, and radiosensitivity in patients with lung adenocarcinoma. (A, B)** The prediction from GEPIA indicated that the expression level of SMAD3 (*p*=0.0021) and p21 (*p*=0.027) were negatively related to overall survival (OS) of lung adenocarcinoma patients in the TCGA database. **(C, D)** The prediction from KMplot indicated that the expression level of SMAD3 (*p*<0.001) and p21 (*p*=0.004) were negatively related to overall survival (OS) of lung adenocarcinoma patients using KMplot. **(E)** Relationship between the level of SMAD3 and p21 of patients with lung adenocarcinoma was analyzed using GEPIA. **(F, G)** Unsupervised univariate analysis using Kaplan-Meier survival analysis of patients with lung adenocarcinoma in our hospital showed that patients with high expression of SMAD3 or p21 both had shorter OS and were associated with poor prognosis. **(H-M)** The representative images of the 119 lung adenocarcinoma patients showed the level of SMAD3 and p21 in the same patient analyzed with tissue microarrays (Magnification×40 and ×200). RS, radiation-sensitive. RR, radiation-resistant.

We investigated the correlation between SMAD3 or p21 expression and the survival of 192 patients in the TCGA database and 1,926 patients in the GEO database using GEPIA and KMplot, respectively, and we found that the expression of both SMAD3 and p21 were significantly negatively correlated with patient survival (both *p* < 0.001). Patients with high expression of SMAD3 or p21 showed a tendency for shorter survival (**Fig. 8A- 8D**). We also used GEPIA to analyze the correlation between the expression of SMAD3 and p21 in TCGA lung adenocarcinoma patients, and found that the level of SMAD3 and p21 are highly positively correlated (R = 0.28, *p* < 0.001) (**Fig. 8E**).

Tissue microarrays was used to investigate the expression of SMAD3 and p21 in the tumor samples of 119 lung adenocarcinoma patients who underwent surgery in our department. Based on unsupervised univariate analyses using Kaplan-Meier survival analysis, we also determined that patients in our department with high expression of SMAD3 or p21 both had shorter OS. We also found that in the same patient the SMAD3-low exprssion patient was with the low expression of p21 and in SMAD3-high patient also was with high expression of p21 (**Fig. 8F, 8G**). We checked all 42 patients who underwent postoperative radiotherapy and their follow-up were completed in December 2015. And then we matched nine pairs patients for gender, age, tumor staging. These matched patients were divided into two groups, 9 of whom were in the radiation-sensitive (RS) group and 9 in the radiation-resistant (RR) group. The RS patients were defined as having experienced early recurrence or metastasis or died from the disease within 3 year., whereas RR patients were defined as having passed the 3-year survival period with no evidence of recurrence or metastasis. The matched patients are listed in the **Table 1**. We used a paired test to investigate the expression of SMAD3 and p21 in these patients and found that patients with postoperative radiotherapy and poor prognoses had high expression of SMAD3 and p21 (**Fig. 8H-8M**). This suggested that high expression of SMAD3 and p21 may be associated with poor postoperative radiotherapy. Our results further confirmed that patients with high expression of SMAD3 and p21 had a poor prognosis.

## Discussion

In this study, we found that knockdown of SMAD3 by two shRNAs inhibited cell growth and promoted the radiosensitivity of three lung adenocarcinoma cell lines. Our results demonstrated that SMAD3 could directly regulate the transcription of p21, which was important in the effects of SMAD3 on cell growth and radiosensitivity *in vitro* and *in vivo*. Furthermore, using the data from thousands of patients in online databases and our department, we determined that the expression levels of SMAD3 and p21 were highly positively correlated with patients' survival as well radiotherapy outcomes. Overall, the data in this study indicated that SMAD3 was a potential indicator of prognosis and radiosensitivity as well as a target for radiotherapy and other treatments of lung adenocarcinoma.

Radiotherapy plays an important role in the treatment of lung adenocarcinoma at present. Therefore, the identification of targets related to the radiosensitivity of lung adenocarcinoma cells is clinically important. Yang et al. recently reported that long noncoding RNA LINC00483 regulates the radiosensitivity of lung adenocarcinoma by interacting with the microRNA-144/HOXA10 signaling axis 12. Jiang et al. demonstrated that RNA interference-mediated gene silencing of cyclophilin A markedly enhanced the radiosensitivity of PAa human lung adenocarcinoma cells *in vitro* and induced G2/M phase cell cycle arrest 13. Dong et al. showed that speckle-type POZ protein affected DNA damage response repair kinetics, apoptosis, and cell cycle checkpoints to regulate the response of lung adenocarcinoma cells to radiation 14. These studies indicate that non-coding RNA or encoding RNA can modulate radiosensitivity through different mechanisms. Changing the radiosensitivity by regulating genes is a potential therapeutic approach for adenocarcinoma patients who need radiotherapy. Although significant research efforts have been made in the study of radiosensitivity of patients, radioresistance still limits the radiotherapy clinical efficient for the treatment of lung adenocarcinoma.

The transcription factor SMAD3 is involved in the regulation of various physiological and pathological processes, including carcinogenesis 15-18. In the present study, we found that knockdown of SMAD3 resulted in significant inhibition of cell growth via p21/cell cycle in lung adenocarcinoma cells and a mouse model. Several studies have reported anti-tumor effects following knockdown of SMAD3. Tang et al. reported that deletion or inhibition of SMAD3 in the tumor microenvironment suppresses tumor growth, invasion, metastasis, and death 19. Qian et al. reported that reduced expression of SMAD3 led to the downregulation of PAX6 mRNA and protein levels along with decreased cell migration, invasion, proliferation, and viability in A549 and HCC827 cells 16. The relationship between SMAD3 and cancer patients' prognosis has been reported. Kim et al. reported that SMAD3 expression was an independent predictor of both shorter disease-free survival and shorter OS in gastric carcinoma patients following curative surgery 20. Zhang et al. suggested that high expression of SMAD3 was associated with poor event-free survival and shorter OS in acute myeloid leukemia patients undergoing chemotherapy 21. Similar to previous studies, we also found that the expression level of SMAD3 was significantly associated with the prognoses of thousands of patients with lung adenocarcinoma. Tang et al. reported that SMAD3+/+ mice developed lung cancer with a 100% mortality rate compared with SMAD3-/- mice 19. This study indicate that targeting SMAD3 may be an effective therapy to protect against cancer progression and increase OS. However, the molecular mechanism by which SMAD3 affects prognosis and OS is not fully understood. Jiang et al. showed that silencing of SMAD3 increased the sensitivity of glioma cells to radiotherapy by interacting with the MRE11-RAD50-NBS1 complex 22. Moreover, knockdown of SMAD3 reduces colony-forming ability in irradiated A549 and MDA-MB-231 cells by abrogating radiation-induced DLX2 expression 23. These results indicate that there may be other mechanisms that also affect SMAD3 and radiosensitivity. However, Li et al. reported that overexpression of SMAD3 increased cell viability and colony formation in human nasopharyngeal carcinoma CNE-2 cells treated with radiotherapy 8, suggesting that SMAD3 may have a variety of effects on radiotherapy in different tumors.

The p21 is a key mediator of numerous genes. Several transcription factors, including SMAD3, regulate the transcription, stability, and cellular localization of p21, thereby regulating its activity 24. However, the exact binding sites of SMAD3 in the p21 promoter are unknown 25-27. Here, we validated the regulatory effect of SMAD3 on p21 and investigated binding sites though bioinformatics analyses and dual luciferase assay. The p21 is an important factor influencing the cell cycle, proliferation, and the effects of radiotherapy. The p21 was initially regarded as a tumor suppressor gene because it is the chief mediator of p53-dependent cell cycle arrest 28. It plays an essential role in inhibition of growth following DNA damage, and overexpression of p21 leads to G1 and G2 or S-phase arrest 28, 29. Conversely, p21 may also function as an oncogene because it can inhibit apoptosis 30. Prabhu et al. reported that overexpression of p21 in human osteosarcoma cells protected against etoposide-induced cell death 31. Zagouri et al. reported that overexpression of p21 was associated with a poor prognosis of breast cancer patients 32. P21 protein is still regarded as a protein with dual behavior, as its expression can be beneficial or disadvantageous in patients 33. P21 protein also acts in response to various forms of cellular stresses 24. Thus, further exploration of its role in lung adenocarcinoma is needed. At present, there is no study regarding the role of SMAD3, p21, and radiotherapy. This study found that SMAD3 regulates radiosensitivity via p21 in three cell lines, an animal model, and clinical patient specimens.

## Conclusions

In summary, we determined that SMAD3 regulated radiosensitivity in lung adenocarcinoma via p21 *in vitro* and *in vivo*. Moreover, the level of SMAD3 and p21 expression was negatively correlated with OS of lung adenocarcinoma patients. SMAD3 may be a potential indicator of the radiosensitivity of patients with lung adenocarcinoma.

## Supplementary Material

Supplementary figures and tables.

## Figures and Tables

**Table 1:** Paired clinic characteristics of 18 patients

Groups	Gender	Age	Stage
Patient 1- RS	Male	53	III
Patient 1- RR	Male	52	III
Patient 2- RS	Male	55	III
Patient 2- RR	Male	54	III
Patient 3- RS	Male	57	III
Patient 3- RR	Male	60	III
Patient 4- RS	Male	61	III
Patient 4- RR	Male	60	III
Patient 5- RS	Female	63	IV
Patient 5- RR	Female	65	IV
Patient 6- RS	Male	66	III
Patient 6- RR	Male	68	III
Patient 7- RS	Male	67	IV
Patient 7- RR	Male	70	IV
Patient 8- RS	Female	68	IV
Patient 8- RR	Female	73	IV
Patient 9- RS	Male	70	IV
Patient 9- RR	Male	74	IV

*RS* radiation-sensitive, *RR* radiation-resistant
