# Supplementary material for: Knockdown of SMAD3 inhibits the growth and enhances the radiosensitivity of lung adenocarcinoma via p21 in vitro and in vivo
Source: Int J Biol Sci. 2020 Jan 30;16(6):1010–22. doi: 10.7150/ijbs.40173 (PMC7053338; doi:10.7150/ijbs.40173)
Supplement: Supplementary file 1 — Supplementary figures and tables. [file ijbsv16p1010s1.pdf]

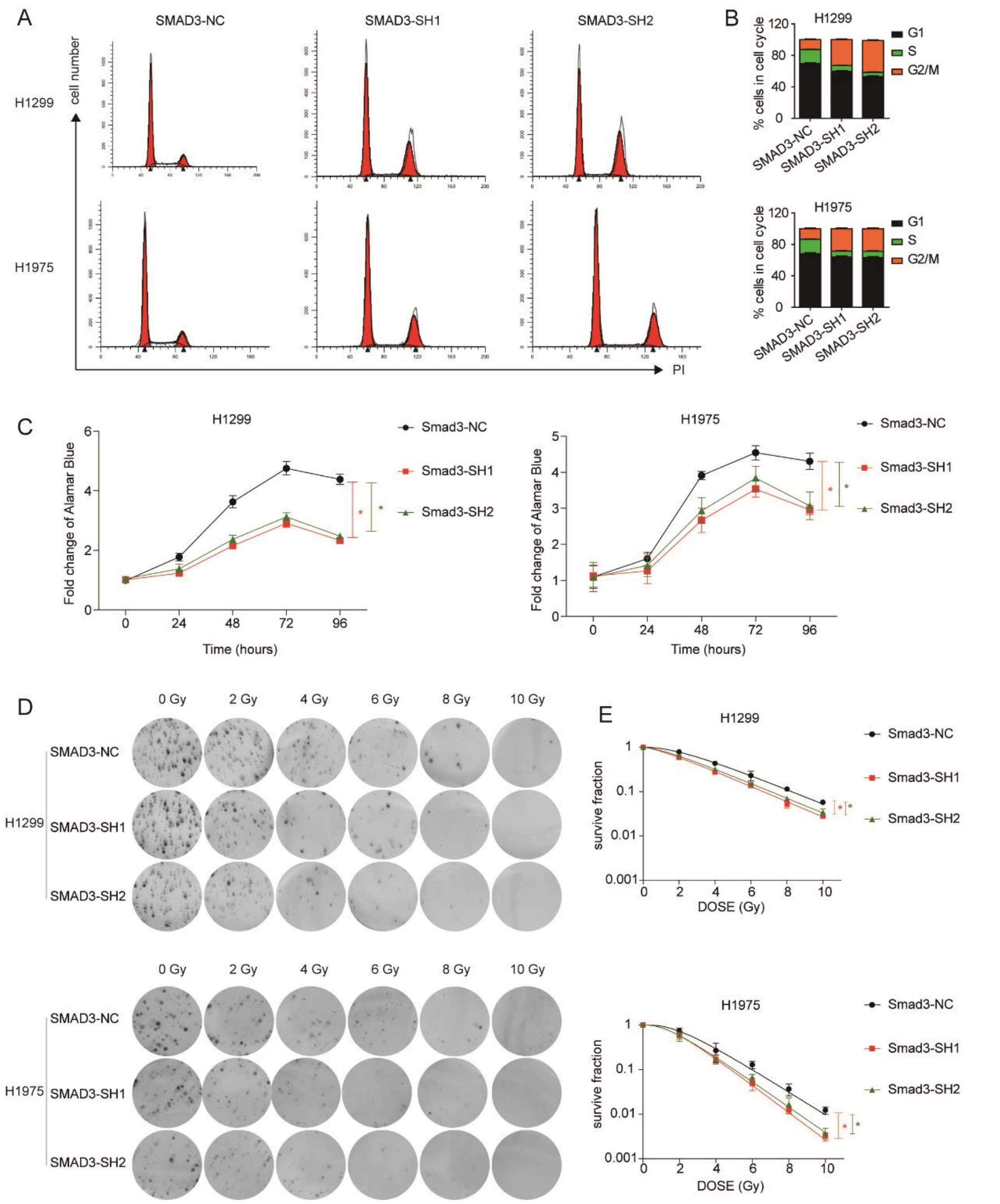

Figure Supplementary 1. **Knockdown of SMAD3 enhanced radiosensitivity and inhibited the proliferation of lung adenocarcinoma cells to irradiation.** (A, B) Knockdown of SMAD3 significantly increased the proportion of G2/M phase in H1299 and H1975 cells detected by flow cytometry. (C) Knockdown of SMAD3 significantly inhibited the proliferation of H1299 and H1975 cells. (D, E) The number colonies of lung adenocarcinoma cells was significantly decreased following knockdown of SMAD3 in H1299 and H1975 cells, as analyzed by clone formation assay and single target multi-shot model curve fitting. Data are

presented as mean ± SEM. $^{*}P < 0.05$, $^{**}P < 0.01$, and $^{***}P < 0.001$. SEM, standard error of mean.

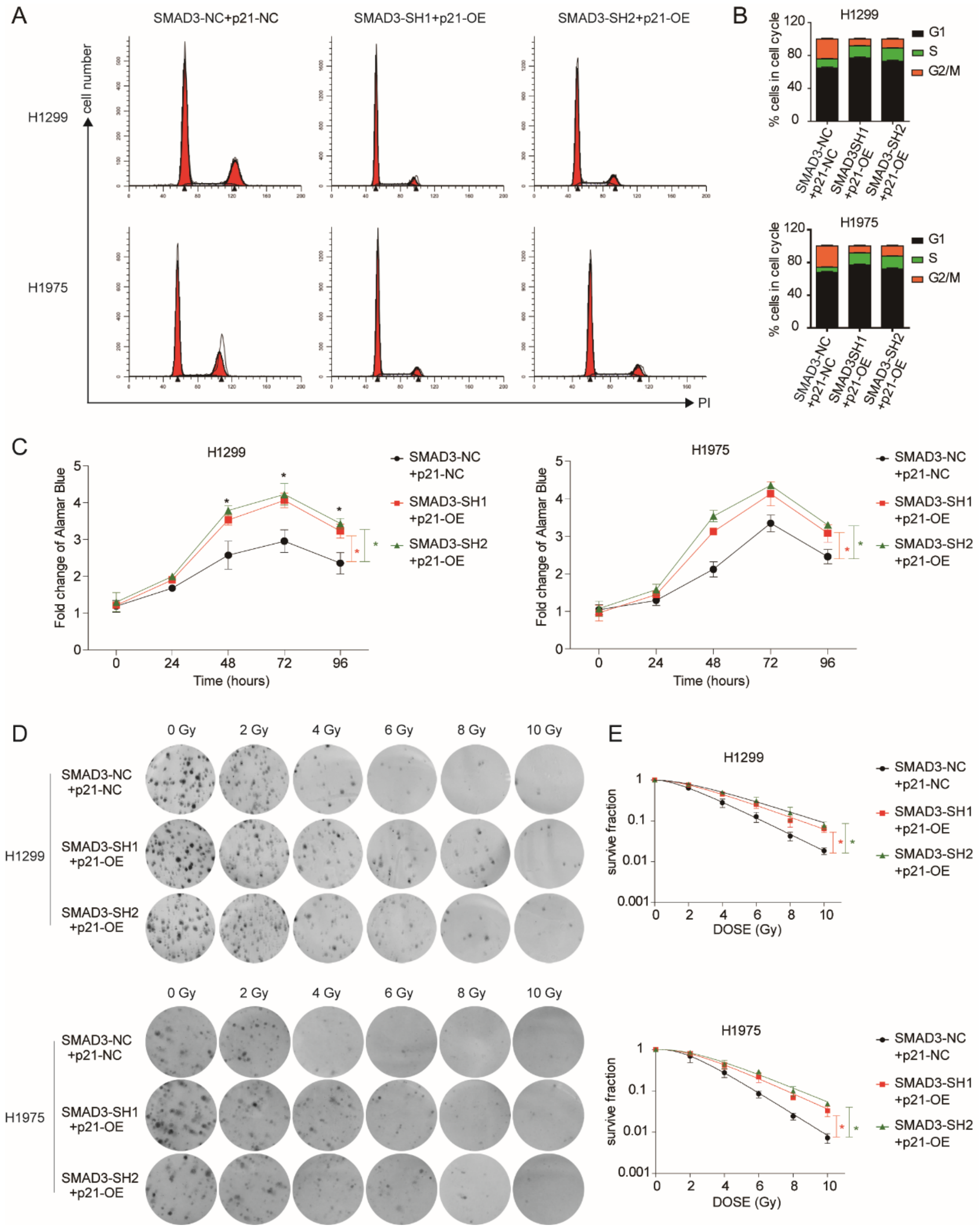

Figure Supplementary 2. Overexpression of p21 partly reversed the effect of knockdown of SMAD3 in lung adenocarcinoma cells. (A, B) Cell cycle analysis demonstrated that overexpression of p21 significantly decreased the percentage of G2/M phase cells in H1299 and H1975 cells infected with shRNA targeting SMAD3 by flow cytometry. (C)

Overexpression of p21 reversed the inhibition of proliferation caused by knockdown of SMAD3 in H1299 and H1975 cells. (D, E) Analysis of clone formation assay by single target multi-shot model curve fitting demonstrated that the number of cell colonies was significantly increased by overexpression of p21 in H1299 and H1975 cells infected with shRNA targeting SMAD3 at the same dose of irradiation. shRNA, short hairpin RNAs. Data are presented as mean ± SEM. $^{*}P < 0.05$, $^{**}P < 0.01$, and $^{***}P < 0.001$. SEM, standard error of mean.

Table Supplementary 1

		5'-3'
negative control of SMAD3 (SMAD3-NC)		TTGACATGCGCATGGCACGCG
short hairpin 1 of SMAD3 (SMAD3-SH1)		CCAGTGCATATGCAATGTATA
short hairpin 2 of SMAD3 (SMAD3-SH2)		CGGCTATATTGGTTTATGTAGT

Table Supplementary 2

primers		5'-3'
β-ACTIN	F	TGACGTGGACATCCGCAAAG
	R	CTGGAAGGTGGACAGCGAGG
SMAD3	F	GATGGAGAGGCTTCCTGATG
	R	TCCGTCTCTTCCTGTGTCCT
p21	F	ATCCCGACTCTTGACATTGC
	R	ACCCTAGACCCACAATGCAG
